# Ubiquitin carboxyl-terminal esterase L1 is not elevated in the serum of concussed rugby players: an observational cross-sectional study

**DOI:** 10.1038/s41598-022-16447-1

**Published:** 2022-07-18

**Authors:** Jazmin O. Harrell, Jessica E. Morgan, Steven D. Beck, Iustin C. Scobercea, Julien S. Baker, Allan Knox, Jorge M. Serrador, Matthew J. Rogatzki

**Affiliations:** 1 Department of Public Health and Exercise Science, Appalachian State University, 1179 State Farm Rd., Boone, NC 28608 USA; 2 Cardio-Renal Physiology Laboratory, Department of Biology, Appalachian State University, North Carolina Research Campus, 150 N Research Campus Drive, Kannapolis, NC 28081 USA; 3 Centre for Health and Exercise Science Research, Hong Kong Baptist University, Kowloon Tong, Hong Kong; 4 Exercise Science Department, California Lutheran University, 60 West Olsen Rd., Thousand Oaks, CA USA; 5 The MARCS Institute for Brain, Behaviour and Development, Western Sydney University, Westmead, NSW Australia; 6 Rehabilitation and Movement Sciences, School of Health Professions, Dept. of Pharmacology, Physiology and Neuroscience, New Jersey Medical School, Rutgers, The State University of New Jersey, Newark, NJ USA

**Keywords:** Biomarkers, Diagnostic markers, Brain injuries

## Abstract

Concussion diagnosis is complicated by a lack of objective measures. Ubiquitin carboxyl-terminal esterase L1 (UCHL1) is a biomarker that has been shown to increase following traumatic brain injury but has not been investigated in concussed athletes on the sideline of athletic events. Therefore, this study was conducted to determine if UCHL1 can be used to aid in sideline concussion diagnosis. Blood was taken via standard venipuncture from a recreationally active control group, a group of rugby players prior to match play (pre-match), rugby players following match-play (match-control), and rugby players after suffering a sport-related concussion (SRC). UCHL1 was not significantly different among groups (p > 0.05) and was unable to distinguish between SRC and controls (AUROC < 0.400, p > 0.05). However, when sex-matched data were used, it was found that the female match-control group had a significantly higher serum UCHL1 concentration than the pre-match group (p = 0.041). Differences were also found in serum UCHL1 concentrations between male and female athletes in the match-control group (p = 0.007). This study does not provide evidence supporting the use of UCHL1 in sideline concussion diagnosis when blood is collected soon after concussion but does show differences in serum UCHL1 accumulation between males and females.

## Introduction

Sport-related concussion (SRC) is defined as a traumatic brain injury induced by biomechanical forces in which any neurological deficits eventually resolve and there is no structural damage to the brain that could be detected with structural neuroimaging^1^. Diagnosis of SRC is extremely complex due to confounding symptoms and lack of clinically objective tests^2^. Furthermore, in a self-efficacy survey of 94 high school and collegiate certified athletic trainers (ATCs), self-efficacy in their clinical assessment of SRC, using common tools such as balance testing, neurocognitive testing, vestibular and oculomotor screening, the Sport Concussion Assessment Tool 3rd edition (SCAT3), etc., was reported to be moderate^3^. However, high self-efficacy in concussion diagnosis is important to ensure the safety of concussed athletes^3^. Including a validated objective blood-based biomarker in the diagnostic criteria of SRC would likely enhance the accuracy of SRC diagnosis and improve self-efficacy among ATCs and other medical practitioners.

Currently, there is no clinically validated biomarker that can be used to aid in SRC diagnosis on the sideline of athletic events^4^. Ubiquitin carboxyl-terminal esterase L1 (UCHL1) is an enzyme primarily found in neurons and is involved with tagging cytosolic proteins for degradation^5^. This enzyme has emerged as a strong biomarker candidate for assessing severe traumatic brain injury (TBI)^6–8^. Research has also shown that UCHL1 is elevated in moderate and mild TBI patients, and can be used to distinguish mild TBI patients requiring computed tomography (CT) imaging^9–13^. In 2018, UCHL1 was approved by the United States of America Food and Drug Administration (FDA) to be used in the assessment of mild TBI in lieu of head CT imaging. Recently, the company Abbott Laboratories (Chicago, IL USA) has developed the FDA approved Abbott's i-STAT™ Alinity™ handheld device which can produce UCHL1 results within 15 min after a plasma sample has been inserted. This technology has been shown to have good sensitivity in predicting acute traumatic intracranial injury^14^. All these advancements make the investigation of UCHL1 as a biomarker that may aid in SRC diagnosis quite lucrative.

In a clinical investigation, Papa et al. found that UCHL1 was highest at enrollment^15^. This study also showed that UCHL1 was able to distinguish TBI patients (most of whom were mild TBI patients) compared to controls at enrollment as well as 4, 8, 12, and 16 h post-injury^15^. A more recent study by Papa et al. found that UCHL1 is detectable less than one hour after mild TBI^12^. Based on these studies it is plausible that UCHL1 could be used to detect SRC on the sideline of an athletic event. A few studies have investigated UCHL1 in athletes who experienced an SRC and found that UCHL1 levels are elevated^16–18^.

Asken et al. compared UCHL1 levels in SRC subjects to baseline and controls with blood drawn 10 h after SRC (median 4.1 h post SRC)^16^. From this study it was found that UCHL1 levels were higher in SRC subjects compared to baseline and controls but not statistically different^16^. However, when data analysis was limited to blood samples obtained less than four hours after injury UCHL1 levels were significantly higher compared to baseline^16^.

Meier et al. compared blood draws from preseason and football player controls to football players experiencing SRC^18^. In this study UCHL1 was found to be significantly higher in SRC subjects at 6 h (median blood collection time point of 2 h post SRC) compared to preseason and football player controls^18^. In a similar study by Meier et al., blood was collected at preinjury baseline, within 6 h post SRC (median 3.8 h. post SRC) and 24 to 48 h post SRC^17^. These data were then compared to matched uninjured football players and non-contact-sport athletes finding that UCHL1 was elevated within 6 h post SRC compared to baseline and both control groups^17^.

Past studies on SRC and mild TBI all point to UCHL1 being a potential biomarker for use in sideline diagnosis based on how rapidly UCHL1 seems to accumulate in the blood. However, there are no studies to date that focus on collecting blood samples on the sideline of athletic events soon after concussion. Therefore, the purpose of this study was to investigate serum UCHL1 by attending rugby tournaments and collecting blood pitchside from concussed and non-concussed rugby players. We hypothesized that UCHL1 would be able to distinguish concussed athletes pitchside.

## Methods

The Appalachian State University (Boone, NC, USA) (IRB #s 20-0033, 19-0232, and 17-0345) and Rutgers University (New Brunswick, NJ, USA) (IRB# Pro20140000411) Institutional Review Board (IRB) for the Protection of Human Subjects approved this study. All data was collected in accordance with guidelines and regulations of both the Appalachian State University and Rutgers University IRBs. Prior to participation, written informed consent was obtained from all subjects. All subjects acknowledged that they cannot be identified via the paper and subjects were fully anonymized following data collection.

### Study population and study design

**Figure 1:**
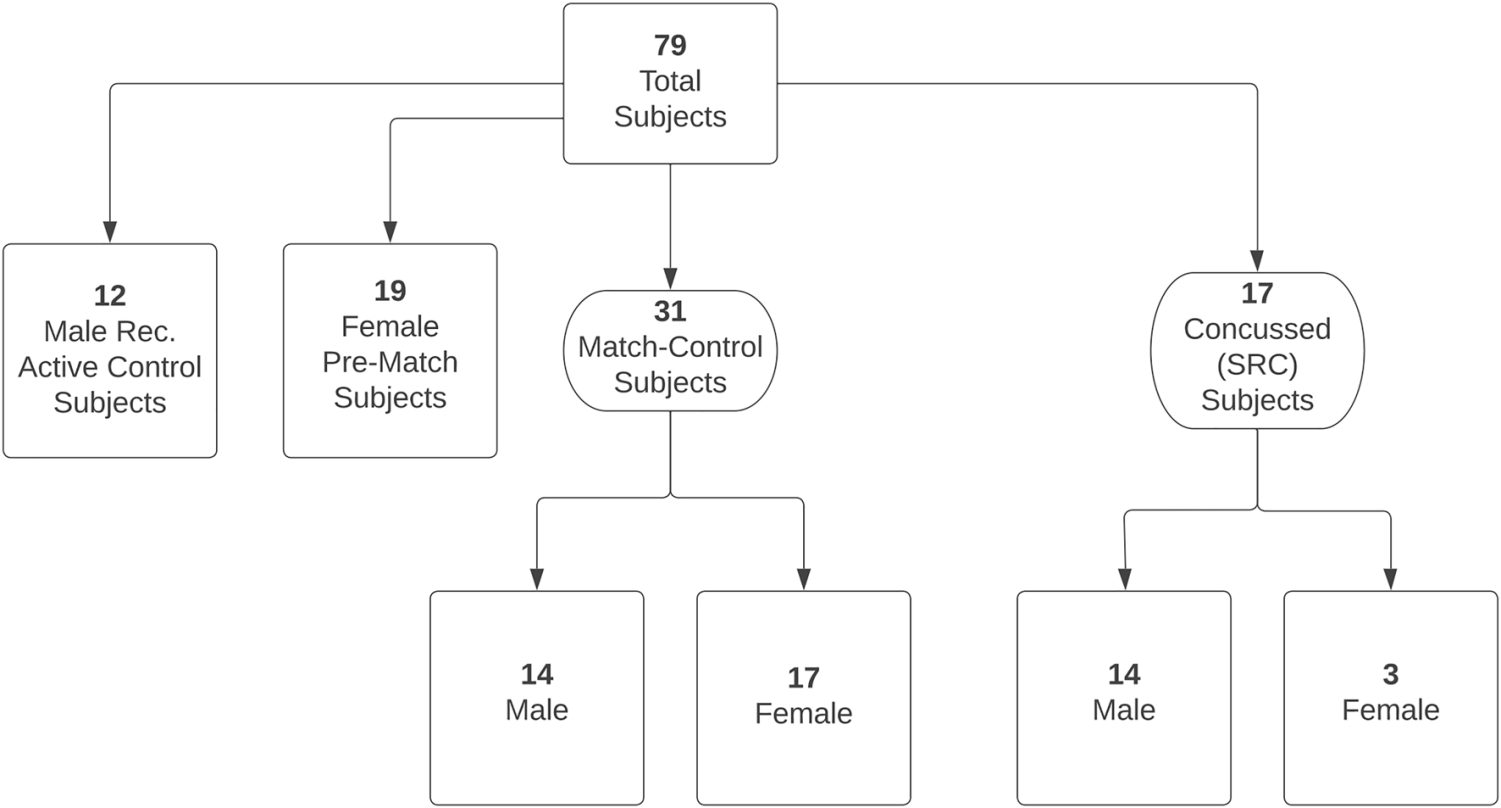
Schematic explanation of subject groups. *Rec.* recreationally, *SRC* sport related concussion.

This study was an observational cross-sectional study with the primary objective of determining if UCHL1 can distinguish among concussed and non-concussed subjects at the sideline of athletic events. Rugby players were separated into pre-match (n = 19), match-control (n = 31) and SRC groups (n = 17) (Fig. 1). Prior to rugby matches, athletes were asked to donate a blood sample for the study. Athletes included in the pre-match group were in-season but had gone seven days without rugby play prior to blood draw. The athletes donating blood were asked to return to the research tent following their match to participate in the match-control group and increase the number of paired samples. There were only females rugby players in the pre-match group because none of the male rugby players agreed to donate a blood sample pre-match despite repeated recruitment. Since no male rugby players volunteered to donate blood pre-match, we included a separate pool of male Caucasians (n = 12) who were of similar age to the majority of match-control male athletes, recreationally active, and not participating in any contact sport to serve as a male control group. These recreationally active controls had not exercised eight hours prior to blood draw. Match-control and SRC athletes were in-season and had not played a match in the seven days prior to participation in the study. Match-control subjects included females (n = 17) and males (n = 14) who were recruited by asking rugby players to participate in the study following their rugby match. None of the rugby athletes participating in the match-control group reported injury of any kind. Subjects in the SRC group included females (n = 3) and males (n = 14) recruited by asking athletes who were diagnosed with concussion by on-site ATCs to participate in the study. None of the SRC subjects experienced loss of consciousness and all were able to give consent. Although ATCs have reported moderate self-efficacy in diagnosing concussion^3^, they are the best tool we currently have for diagnosing concussion and is the reason their diagnosis was relied upon for this study. Subjects in all groups were asked about their concussion history (number of past concussions), years of rugby play, age, body mass, and all subjects reported they had never been diagnosed with any central nervous system dysfunction such as epilepsy, cerebral palsy, or multiple sclerosis. Demographic characteristics of each group can be found in Table 1.

**Table 1:** Descriptive statistics for each group with separated male and female data. Subjects were all Caucasian.

Group	Sex (n)		Age (yrs.)		Mass (kg)		Concussion Hx (n)		Years of play (yrs.)	
	Male	Female	Male	Female	Male	Female	Male	Female	Male	Female
Rec. active ctrl (n = 12)	12	0	20.3 (1.5)	N/A	68.4 (6.6)	N/A	0 (0)	N/A	0 (0)	N/A
Pre-match (n = 19)	0	19	N/A	20.2 (4.6)	N/A	73.3 (15.0)	N/A	0 (0, 1.5)	N/A	1.0 (1.0, 2.0)
Match-ctrl (n = 31)	14	17	37.5 (24.3, 49.5)	20.5 (2.9)	93.6 (14.0)	72.6 (14.4)	2.0 (1.0, 2.8)	0 (0, 1.0)	11.5 (5.8, 26)	1.0 (1.0, 3.0)
SRC (n = 17)	14	3	24.5 (22.0, 27.5)	18.3 (0.5)	88.5 (9.4)	63.6 (6.7)	0.5 (0, 1.8)	0 (0)	7.0 (2.5, 9.5)	1.0 (1.0, 1.5)

All data reported as mean (SD) except when the SD is greater than 1/3 of the mean. In these cases data are reported as median (Q1, Q3). Rec. Active Ctrl = Recreationally active control group, Match-Ctrl = Match-Control group, SRC = Sport-related Concussion group, Hx = history, SD = standard deviation, Q1 = 1st quartile, Q3 = 3rd quartile.

### Blood collection and processing

One four milliliter blood sample from subjects in all groups was obtained from a prominent vein in the antecubital space by standard venipuncture. Blood collection was performed in a laboratory setting for the recreationally active control group and occurred in a research tent pitchside for the pre-match, match-control and SRC groups. Our goal was to collect blood less than 60 min from the end of the match in the match-control subjects and less than 60 min from the impact that caused concussion in the SRC group. Blood was drawn into a silicone-coated serum tube and allowed to clot at ambient temperature for at least 30 min but no more than 60 min. After the ambient temperature incubation period the blood was centrifuged using a StatSpin Express 3 portable centrifuge (Beckman Coulter, Inc., Brea, CA USA) for five minutes at 5600 revolutions per minute (2685 relative centrifugal force) to separate the serum from red blood cells. Serum was then removed using a pipettor and placed in a cryotube which was placed in a cryobox and then stored in a −80 °C portable freezer (model ULT25NEU, STIRLING ULTRACOLD A division of Global Cooling, Inc., Athens, OH USA) until the sample could be placed in a permanent −80 °C freezer. The samples remained frozen at −80 °C until biomarker analysis.

### Biomarker analysis

Serum UCHL1 concentration was measured using human UCHL1 ELISA-96-well plate assay kits (OKEH01247, Aviva Systems Biology Corporation, San Diego, CA USA). The detection range of the assays were 78–5,000 pg mL^−1^, with a limit of detection (LOD) less than 34 pg mL^−1^. A linear regression analysis was used to formulate a standard curve for each assay. The average intra-assay coefficient of variation was 16.3% and the inter-assay coefficient of variation was 6.66%.

Analysis of all standards and samples were performed in duplicate. All values found to be below the detection range of the assay were reported to be one-half the LOD. In addition, all values found to be below the blank value were reported as undetectable. Absorbance for all five assays were read at 450 nm using an Eon spectrophotometer (BioTek Instruments, Inc., Winooski, VT USA).

### Statistical analyses

G*power analysis (G*Power 3.1.9.6)^19^ using previously published data^16,20^ determined that 12 subjects in each group were needed to obtain a power of 0.80 and effect size of 0.5.

A Kruskal–Wallis non-parametric test was used to compare medians of UCHL1, age, body mass, concussion history, and years of rugby play among groups due to unequal sample sizes and a lack of normal distribution as examined using a Shapiro–Wilk normality test. Partial eta squared (η^2^) was used to calculate effect size. The area under the receiver operating characteristic curve (AUROC) was calculated to determine the diagnostic accuracy of UCHL1 to distinguish between groups. Since Asken et al. found differences in resting UCHL1 concentration between males and females^21^ we decided to perform a separate statistical analysis comparing male data only and female data only among the protocols (i.e. pre-match females vs. match-control females, pre-match females vs. SRC females, match-control females vs. SRC females, recreationally active males vs. match-control males, recreationally active males vs. SRC males, and match-control males vs. SRC males). We also performed a separate statistical analysis comparing male and female data within the match-control and SRC groups (i.e. match-control females vs. match-control males and SRC females vs. SRC males). Since the Shapiro–wilk normality test also showed a lack of normality for the male and female match-control and SRC data, a two-sided Mann–Whitney U test was used to compare medians between sexes and a Cohen’s d (d) test was used to calculate effect size. A sensitivity analysis was performed to determine if age affects UCHL1 levels since age has been shown to affect the appearance of certain brain injury biomarkers, specifically neurofilament light (NfL)^22^, and because there was a statistical difference between the pre-match group and both the match-control and SRC groups. The sensitivity analysis removed data from subjects aged 40 years or older based upon outliers considered as a Z score less than − 1.5 or greater than 1.5 (a conservative Z score for determining outliers was used as using the typical Z score of 3 and − 3 would have only removed two subjects), which ended up being a total of 9 subjects. We used this same sensitivity analysis method to detect outliers in the time of blood draw in the match-control and SRC groups since there was a broad range in blood collection times. A sensitivity analysis was also conducted for serum UCHL1 concentration for each group. However, we used the more common Z score of − 3 and 3 as a cutoff value for UCHL1 concentration outliers. Statistical significance was determined at p < 0.05. All statistical analyses were generated using Statistical Package for the Social Sciences (SPSS) version 27 (SPSS Inc., Chicago, IL USA).

### Ethics approval and consent to participate

The Appalachian State University (Boone, NC, USA) (IRB #s 20-0033, 19-0232, and 17-0345) and Rutgers University (New Brunswick, NJ, USA) (IRB# Pro20140000411) Institutional Review Board (IRB) for the Protection of Human Subjects approved this study.

## Results

The study sample consisted of 79 total participants. We recruited 19 individuals pre-match, all of which were female. Post-match, 31 individuals (14 male and 17 female) consented to giving their blood, among which 5 female individuals also provided their blood pre-match. The recreational active male control group consisted of 12 individuals. Finally, blood sampling for UCHL1 was available for 17 athletes (14 male and 3 female) with SRC. Blood was collected an average of 42.3 min, (median = 16.0 min, Q1 = 10.0 min, Q3 = 45.0 min) post-match in the match-control group and blood was collected an average of 102.3 min (median = 37.0 min, Q1 = 10.0, Q3 = 156.0 min) post-concussion in the SRC group.

### Demographic comparisons

In comparing demographic data among groups it was found that age was significantly greater in the match-control (median = 22.0 years, Q1 = 19.5 years, Q3 = 27.5 years, p = 0.010, η^2^ = 0.117) and SRC (median = 24.0 years, Q1 = 20.0 years, Q3 = 26.0 years, p = 0.022, η^2^ = 0.117) groups compared to the pre-match group (median = 19 years, Q1 = 18.5 years, Q3 = 20.0 years), but no other differences were found among groups (p > 0.05).

When comparing body mass among groups it was found that the SRC group (median = 87.3 kg, Q1 = 75.0 kg, Q3 = 90.9 kg) had a significantly greater body mass than the recreationally active control group (median = 68.0 kg, Q1 = 63.1 kg, Q3 = 72.9 kg) (p = 0.026, η^2^ = 0.138), but no other differences among groups (p > 0.05, η^2^ = 0.138) were found. Concussion history (number of past concussions) was significantly different between the match-control group (median = 1.0, Q1 = 0.0, Q3 = 2.0) and recreationally active control group (median = 0.0, Q1 = 0, Q3 = 0) (p = 0.002, η^2^ = 0.062), with no differences among any of the rugby groups (p > 0.05). Similarly, years of rugby play were all significantly greater in the rugby groups compared to the non-athlete group (p ≤ 0.002, η^2^ = 0.140), with no differences among the rugby groups (p > 0.05). Table 2 shows the comparison of demographic data among groups.

**Table 2:** Descriptive statistics for combined sex data.

Group	Age (yrs.)	Mass (kg)	Concussion Hx (n)	Years of play (yrs.)
Rec. active ctrl (n = 12)	20.5 (19.5, 21.3)	68.0 (63.1, 72.9)	0 (0.0, 0.0)	0.0 (0.0, 0.0)
Pre-match (n = 19)	19.0 (18.5, 20.0)	70.5 (63.6, 78.4)	0.0 (0.0, 1.5)	1.0 (1.0, 2.0)*
Match-ctrl (n = 31)	22.0 (19.5, 27.5)^+^	81.8 (69.3, 96.1)	1.0 (0.0, 2.0)*	3.0 (1.0, 10.0)*
SRC (n = 17)	24.0 (20.0, 26.0)^+^	87.3 (75.0, 90.9)*	0.0 (0.0, 1.0)	4.0 (2.0, 8.0)*

Subjects were all Caucasian.

All data reported as median(Q1, Q3).

Rec. Active Ctrl = Recreationally active control group, Match-Ctrl = Match-Control group, SRC = Sport-related Concussion group, Hx = history, Q1 = 1st quartile, Q3 = 3rd quartile. * = statistically different compared to recreationally active control group (p < 0.05), ^+^ = statistically different compared to pre-match (p < 0.05).

### Comparisons of UCHL1 concentration

Descriptive statistics of UCHL1 data used for analysis among all protocols can be seen in Table 3. There were no significant differences in serum UCHL1 concentration among all four groups (p = 0.186, η^2^ = 0.138). UCHL1 concentration was significantly higher in the match-control females (median = 124.3 pg mL^−1^, Q1 = 65.5 pg mL^−1^, Q3 = 261.8 pg mL^−1^) compared to the pre-match group (median = 17.0 pg mL^−1^, Q1 = 17.0 pg mL^−1^, Q3 = 73.6 pg mL^−1^) (p = 0.041, η^2^ = 0.080) but there were no differences in males among groups (p = 0.089, η^2^ = 0.303). Match-control female UCHL1 concentration (median = 124.3 pg mL^−1^, Q1 = 65.5 pg mL^−1^, Q3 = 261.8 pg mL^−1^) was significantly greater than match-control male UCHL1 concentration (median = 8.5 pg mL^−1^, Q1 = 0.0 pg mL^−1^, Q3 = 17.0 pg mL^−1^) (p = 0.002, d = 0.590), but there were no differences between the recreational active control group (median = 263.7 pg mL^−1^, Q1 = 0.0 pg mL^−1^, Q3 = 822.1 pg mL^−1^) and pre-match group (median = 17.0 pg mL^−1^, Q1 = 17.0 pg mL^−1^, Q3 = 73.6 pg mL^−1^) (p = 0.236, d = 1.148) or between female (median = 17.0 pg mL^−1^, Q1 = 8.5 pg mL^−1^, Q3 = 161.3 pg mL^−1^) and male (median = 17.0 pg mL^−1^, Q1 = 0.0 pg mL^−1^, Q3 = 17.0 pg mL^−1^) UCHL1 concentration in the SRC group (p = 0.591, d = 0.752). Descriptive statistics comparing UCHL1 concentration within sex-matched data and between male and female data can be seen in Table 3.

**Table 3:** Serum ubiquitin carboxyl-terminal esterase L1 (UCHL1) concentration among groups and between sexes.

Group	Male serum UCHL1 concentration (pg mL^−1^)	Female serum UCHL1 concentration (pg mL^−1^)	Sex combined serum UCHL1 concentration (pg mL^−1^)
Rec. active control group (n = 12 males)	263.7 (0.0, 822.1)	N/A	263.7 (0.0, 822.1)
Pre-match group (n = 19 females)	N/A	17.0 (17.0, 73.6)^+^	17.0 (17.0, 73.6)
Match-control group (n = 14 males and 17 females)	8.5 (0.0, 17.0)	124.3 (65.5, 261.8)*	54.0 (0.0, 197.0)
SRC group (n = 14 males and 3 females)	17.0 (0.0, 17.0)	17.0 (8.5, 161.3)	17.0 (0.0, 17.0)

All subjects were Caucasian. Values are presented as median (Q1, Q3). Rec. = Recreationally, SRC = Sport-related Concussion group, Q1 = 1st quartile, Q3 = 3rd quartile. * = Significantly different compared to male match-control group (p = 0.002), ^+^ = Significantly different compared to female match-control group (p = 0.041).

### Diagnostic accuracy

The AUROC of UCHL1 comparing all groups can be found in Table 4. All comparisons show that UCHL1 did not distinguish between groups in this study (AUROC < 0.400, p > 0.05). We also compared sex-matched groups (Table 5) and found significance between the pre-match and female match-control group (AUROC = 0.745, p = 0.012). However, comparison of all other sex-matched groups showed that UCHL1 did not distinguish between groups (AUROC < 0.600, p > 0.05).

**Table 4:** Area under the receiver operating characteristic curve (AUROC) comparing the different groups using serum.

Group comparison	AUROC (95% CI) for UCHL1
Pre-match vs. rec. active control	0.371 (0.124–0.617), p = 0.232
Match-control vs. rec. active control	0.378 (0.154–0.601), p = 0.218
Match-control vs. pre-match	0.319 (0.140–0.499), p = 0.070
SRC vs. rec. active control	0.309 (0.088–0.530), p = 0.084
SRC vs. pre-match	0.373 (0.186–0.560), p = 0.194
SRC vs. match-control	0.363 (0.202–0.524), p = 0.121

Ubiquitin carboxyl-terminal esterase L1 (UCHL1) concentration. Values are presented as AUROC (95% confidence interval), p-value (p).

Rec. = recreationally; SRC = sport-related concussion.

**Table 5:** Area under the receiver operating characteristic curve (AUROC) comparing sex-matched groups using serum.

Group comparison	AUROC (95% CI) for UCHL1
Pre-match vs. female match-control	0.745 (0.574–0.915), p = 0.012*
Pre-match vs. female SRC	0.474 (0.041–0.907), p = 0.886
Females SRC vs. female match-control	0.371 (0.000–0.758), p = 0.477
Rec. active control vs. male match-control	0.291 (0.092–0.489), p = 0.060
Rec. active control vs. male SRC	0.314 (0.110–0.518), p = 0.094
Male SRC vs. male match-control	0.521 (0.308–0.735), p = 0.844

Ubiquitin carboxyl-terminal esterase L1 (UCHL1) concentration. Values are presented as AUROC (95% confidence interval), p-value (p), Rec. = recreationally, SRC = Sport-related concussion, * = significantly different (p = 0.012).

### Sensitivity analysis

Since age was shown to be a confounding variable in other brain-based biomarkers^22^ we removed nine subjects over the age of 40 and re-ran the statistics. Removal of these data did not affect statistical analysis so these subjects were left in the final data pool. A sensitivity analysis was also conducted on the time of blood collection in the match-control and SRC groups resulting in the removal of one subject from the match-control group and two subjects from the SRC group. Removal of these data points did not alter the statistical outcome so these subjects were left in the final data pool. The sensitivity analysis conducted on serum UCHL1 concentrations found one data point in the match-control female group (UCHL1 concentration = 2805.5 pg mL^−1^) to be an outlier. Upon further analysis of this subject’s demographic data and rugby match notes, we could not find a logical reason to disregard this subject’s data from analysis. However, it is important to note that when this data point was removed from analysis, the statistical difference between the pre-match and female match-control group is no longer statistically significant with a p-value of 0.069 compared to 0.041 when the data point is left in the data set. However, removal of this data point did not change the lack of significance among the sex combined groups nor did it affect significance, or lack thereof, for the AUROC of UCHL1.

## Discussion

This investigation sought to determine if serum UCHL1 concentration from blood collected pitchside could distinguish among concussed rugby players and rugby players who played in a match but did not suffer a concussion, rugby players prior to match play, or recreationally active non-contact sport athletes. The data showed no significant differences in serum UCHL1 concentration among the four experimental groups and AUROC showed that UCHL1 concentration did not distinguish among groups. A large reason no differences were found among groups was due to the high variability of UCHL1 concentration among our subjects. High variability in serum UCHL1 concentration has been previously reported^20^ and may make it difficult to use UCHL1 as a biomarker of concussive injury.

Previous research has reported that athletes experiencing SRC did not have significantly higher UCHL1 levels compared to baseline in serum collected 10 h post-concussion^16^. However, when data analysis was limited to blood samples obtained less than four hours after injury, UCHL1 levels were significantly higher compared to baseline^16^. Our study collected blood samples a median of 37 min after concussion and may not have allowed for significant UCHL1 accumulation, thereby causing our results to not be significant among groups. Although not our primary hypothesis, we postulated that serum UCHL1 concentration may be higher in the match-control group compared to the pre-match and recreationally active group due to subconcussive injury. Subconcussive head injury may occur due to acceleration of the body, torso, or head and may occur during contact sport, such as rugby, despite lack of clinical concussion symptoms^23^. After analysis, it was found that the match-control subjects in our study did not have a significantly greater serum UCHL1 concentration than the recreationally active control group or the pre-match group. This finding is in agreement with a previous study in football players, in which serum UCHL1 concentration was not found to correlate with the number of sub-concussive head hits^24^. This study also agrees with our findings in that UCHL1 levels were unable to distinguish among post-game football players, emergency room patients who experienced mild traumatic brain injury, and healthy controls^24^. Although these studies agree with our findings, suggesting UCHL1 to be an unreliable biomarker of brain injury, other sports-related studies have shown more promise for UCHL1 as a biomarker of brain injury. For example, serum UCHL1 levels were reported to be significantly greater between football players experiencing high-acceleration head impacts compared to those not experiencing high-acceleration head impacts^25^. In our study, we did not separate rugby players based on the number or magnitude of head impacts so it is possible that our match-control group consisted of players experiencing a low magnitude and quantity of hits, and therefore a lack of damage to neurons which would have resulted in elevated serum UCHL1 concentrations. Two other studies investigating concussed football players found that serum UCHL1 concentration was significantly elevated post-concussion compared to baseline with a fair ability to distinguish between SRC and contact athlete controls or non-athlete controls^17,18^. Blood in these two studies was collected 6 h (median of 3.8 h^17^ and median of 2 h^18^) post-concussion and may be the critical difference in their findings compared to ours. In our study blood draws were taken on average 102.29 min (1 h and 42 min) with a median time of 37 min post-SRC. Furthermore, in trauma patients experiencing mild to moderate TBI, although the presence of UCHL1 was found in serum one-hour post injury, it was not until 8-h post-injury that UCHL1 levels were found to peak^15^. Due to the timing of our blood draws, we may not have seen large increases in serum UCHL1 concentration due to the kinetics of UCHL1 appearing in the blood. In a one-compartment kinetic model developed by Aziz et al. (2021), it was determined that UCHL1 levels likely reach a maximum appearance in the blood at 8 h following concussion, while gradually increasing up to that point^26^. Brophy et al. investigated the kinetics of UCHL1 levels in severe traumatic brain injury serum obtained 6 h after injury up to 7 days post-injury and found maximum UCHL1 concentration in serum appeared approximately 9 h after severe traumatic brain injury^6^. Our findings seem to support both of these kinetic models predicting that more time may need to elapse before UCHL1 is able to accumulate in the blood and indicate brain injury. Kinetics of UCHL1 appearance in the blood may point to difficulty in using UCHL1 levels to diagnose concussion at the sideline of athletic events when diagnosis must be made almost immediately post-injury to protect the athlete. Based on our results, and those of other studies on concussed athletes, UCHL1concentration does not appear to be a viable a biomarker in diagnosing SRC at the sideline of athletic events as it has shown to be in mild or moderate traumatic brain injury patients^10^. Although, similar results to our findings have also been shown when examining blood concentrations of UCHL1 in adult and pediatric trauma patients.

A study investigating adult and pediatric trauma patients between 4 and 180 h post injury did not find significant differences in serum UCHL1 concentration between concussion patients and head trauma control or body trauma control patients^27^. Another study investigating brain injury in children under the age of 15 years found that serum UCHL1 concentration in blood collected within 24 h of head injury was significantly greater in subjects with moderate and severe TBI compared to healthy controls but no difference in UCHL1 concentration was found between mild TBI subjects and healthy controls^28^. In a study investigating plasma UCHL1 concentration in blood samples collected between 2 and 6 h post injury in children with mild head injury, it was found that plasma UCHL1 levels were below the detection limit of the assay (0.78 ng/mL) for all subjects^29^. Based on these studies it may be possible that concussion does not necessarily cause enough neurotrauma to significantly increase UCHL1 levels, and UCHL1 concentration may be better at indicating more severe types of brain injury than typically seen during athletic events.

Very little research has compared UCHL1 levels in male and female athletes. Asken et al. (2018) found that male athletes tend to have higher median serum baseline UCHL1 levels than female athletes^21^. Our study agrees with this finding as values for the pre-match group (solely composed of females) had a lower, although not statistically significant, UCHL1 concentration than the recreationally active control group (solely composed of males). However, the lack of statistical significance may be due to the male group being composed of recreationally active individuals not participating in a contact sport whereas the female group was composed of in-season rugby players who may have had elevated UCHL1 levels. In addition to this difference in sexes, we found statistically significant sex differences within the match-control group. In this group the data was inverse to that of the baseline data, with females having higher UCHL1 levels than males. Females may have had higher UCHL1 levels post-match compared to males due to differences in neck size and strength between females and males. Research has shown that females are at greater risk for sustaining concussion than males, possibly due to female athletes having smaller and weaker neck muscles^30^. Females have also shown significantly greater head-neck segment peak angular acceleration and displacement than males when subjected to external loads to simulate real-life sport situations^31^. Because of this, the subconcussive impacts experienced during a rugby match may cause more neuronal damage in female athletes compared to male athletes, and therefore greater serum UCHL1 concentration. However, since neck girth or strength were not investigated in the current study, this hypothesis is purely speculative.

Because of these sex differences, we decided to compare sex-match data among the different groups in our study. After doing this, we found that the female match-control group had significantly higher serum UCHL1 concentration than the pre-match group and the AUROC was also significant for distinguishing between the female match-control group and pre-match group. However, there were still no significant differences among the male matched data groups. These comparisons seem to support the hypothesis that subconcussive hits may result in more neurotrauma in female athletes compared to their male counterparts.

There are several limitations in our study. Our sample population was small and lacked matching in age, sex, years of play, and concussion history. Our data also lacked paired data from pre- to post-match and pre-match to post-SRC. Paired data would have been beneficial in determining if UCHL1 levels could distinguish match-play and concussion within individuals. Another limitation is that we only obtained blood samples at one time point and the time points had large variation from 5 to 303 min post-match and 6 min to 480 min post-concussion. It would have been ideal to collect initial blood samples at the same time post-match and post-concussion, along with analyzing how UCHL1 accumulated in the serum at later time points. This would also have made our data more comparable to other studies. Relying on on-site ATCs not involved in the study to diagnose concussion is also a limitation, as ATCs have reported moderate efficacy in diagnosing concussion. This is a necessary limitation as current SRC diagnosis largely relies on athlete assessment by ATCs. A lack of concussion assessment on the match-control subjects is also a limitation since it is possible that some of the subjects in the match-control group were concussed even though they reported not having any symptoms of concussion.

To the best of our knowledge this is the first study focused on analyzing serum UCHL1 concentration from blood samples obtained soon after SRC. No difference in serum UCHL1 concentration was found in any of the combined sex groups, suggesting that UCHL1 levels may not be a viable biomarker for sideline SRC diagnosis at early timepoints. However, as indicated by other studies, UCHL1 levels may be useful in diagnosing SRC at later time points, specifically 8 h or more after concussion. Interestingly, sex differences in UCHL1 concentration were found, indicating the need for sex-matched groups in future studies investigating UCHL1 levels. In order for biomarkers to be used in sideline concussion diagnosis future research must focus on collecting blood within one-hour post injury along with later time points. As seen with past studies investigating UCHL1 concentration, blood collection at later time points such as 4–8 h post-injury may be useful in distinguishing concussed from non-concussed subjects but, as indicated in our study, blood collected soon after injury may not provide the same results.

## Data Availability

The datasets used and analyzed during the current study are available from the corresponding author upon reasonable request.
